# *Notes from the Field:* An Outbreak of *Escherichia coli* O157:H7 Infections Linked to Romaine Lettuce Exposure — United States, 2019

**DOI:** 10.15585/mmwr.mm7018a3

**Published:** 2021-05-07

**Authors:** Connor Hoff, Jeffrey Higa, Kane Patel, Ellen Gee, Allison Wellman, Jeff Vidanes, April Holland, Varvara Kozyreva, Jonathan Zhu, Mia Mattioli, Alexis Roundtree, Kenai McFadden, Laura Whitlock, Matthew Wise, Laura Gieraltowski, Colin Schwensohn

**Affiliations:** ^1^Division of Foodborne, Waterborne, and Environmental Diseases, National Center for Emerging and Zoonotic Infectious Diseases, CDC; ^2^Oak Ridge Institute for Science and Education, Oak Ridge, Tennessee; ^3^California Department of Public Health; ^4^Food and Drug Administration, College Park, Maryland; ^5^Placer County Health and Human Services, Auburn, California; ^6^Eagle Medical Services, LLC, San Antonio, Texas.

On September 16, 2019, PulseNet, the national molecular subtyping network for foodborne disease surveillance, reported a multistate cluster of seven *Escherichia coli* O157:H7 infections from California (five), Oregon (one), and Pennsylvania (one). Isolates from cases of human illness were sequenced and then analyzed using core-genome multilocus sequence typing (cgMLST); the isolates were closely related within three allele differences (*1*). Federal, state, and local officials initiated a multistate outbreak investigation to identify the source and prevent additional illnesses.

State and local investigators interviewed patients to assess common food, restaurant, and grocery store exposures. Once investigators identified leafy greens as a suspected source of infection, a focused questionnaire was developed to collect detailed information about patients’ restaurant and leafy greens exposures. By September 30, 2019, the California Department of Public Health (CDPH) identified five of six patients who reported eating at one of four locations of a national restaurant chain, including two unrelated patients who reported eating at the same restaurant chain location. All patients reported consuming salads containing romaine lettuce.

A case was defined as isolation of *E. coli* O157:H7 with the cgMLST profile matching the outbreak strain from an *E. coli* O157:H7 infection during July–October 2019. In total, PulseNet identified 23 cases in 12 states: California (eight), Arizona (three), Illinois (two), Pennsylvania (two), and one each in Florida, Georgia, Maryland, Nevada, New York, North Carolina, Oregon, and South Carolina. Illness onset dates ranged from July 12 to September 8, 2019. Patient ages ranged from 3 to 81 years (median = 43 years); 82% were female. Sixty percent of patients were hospitalized, and no deaths were reported. This activity was reviewed by CDC and was conducted consistent with applicable federal law and CDC policy.*

Among patients with available information, 16 of 17 reported eating leafy greens, and 11 (85%) of 13 reported eating romaine lettuce in the week before becoming ill. This percentage was higher than the 47% (p<0.02) of persons who, in the 2006–2007 survey of healthy persons, reported eating romaine lettuce during the week before they were interviewed (*2*). Among the 11 patients who reported consuming romaine lettuce, six (five from California and one from Illinois) reported eating romaine lettuce in salads at one of five locations of the national restaurant chain; the remaining five patients reported eating it at other restaurants or purchasing it from grocery stores.

CDPH and the Food and Drug Administration (FDA) conducted a traceback investigation to determine the source of romaine lettuce supplied to the reported restaurant chain locations in California. The traceback identified two farms in California as common sources of romaine lettuce for these locations. FDA, CDPH, California Department of Food and Agriculture, and CDC initiated farm-level investigations on October 10, 2019. Investigators conducted an environmental assessment and collected soil, animal droppings, and water samples for laboratory testing; *E. coli* O157:H7 was not detected. A public warning was not issued because all romaine lettuce harvested from the two farms was past its shelf life, no longer available for purchase, and unlikely to be in persons’ homes, indicating that there was no ongoing risk to the public.

Recent Shiga toxin-producing *E. coli* outbreaks associated with romaine lettuce highlight the continued food safety challenges associated with consumption of fresh leafy greens (*3*,*4*). Once epidemiologic and traceback data indicated that romaine lettuce from a specific location was the likely source of this outbreak, a field investigation was rapidly initiated, including an environmental assessment to identify possible sources and routes of contamination. Although the outbreak strain was not identified during the field investigations, on-farm investigations are an important component in understanding how a food could have become contaminated and in defining potential approaches to prevent similar contamination events in the future. Preventing contamination at the farm level is important because romaine lettuce is often consumed raw, and washing can remove some but not all harmful bacteria.

## Footnotes

* 45 C.F.R. part 46.102(l)(2), 21 C.F.R. part 56; 42 U.S.C. Sect. 241(d); 5 U.S.C. Sect. 552a; 44 U.S.C. Sect. 3501 et seq.
